# Homologous recombination deficiency test validation in patients with high-grade advanced ovarian cancer

**DOI:** 10.3389/fmolb.2025.1524594

**Published:** 2025-02-11

**Authors:** Angelica Nogueira Rodrigues, Andreza Karine de Barros Almeida Souto, Diocésio Alves Pinto de Andrade, Larissa Müller Gomes, Sandra Satie Koide, Renata de Godoy e Silva, Bruno Batista de Souza, Juliana Doblas Massaro, Andréia Cristina de Melo, Andrea Morais Borges, Camila Giro, Carlos Augusto Vasconcelos de Andrade, Cesar Martins da Costa, Daniel Luiz Gimenes, Eduardo Caminha Bandeira de Mello, Fernanda Cesar de Oliveira, Frederico Müller de Toledo Lima, Gabriel Lima Lopes, Gustavo de Oliveira Bretas, Gustavo Guerra Jacob, Herika Lucia da Costa Silva, Juliana Ferrari Notaro, Lara Ladislau Alves, Marcos Veloso Moitinho, Mirian Cristina da Silva, Roberto Abramoff, Thais Amaral da Cunha Rauber, Rodrigo Dienstmann, Fernanda Christtanini Koyama

**Affiliations:** Oncoclínicas&Co, São Paulo, Brazil

**Keywords:** ovarian cancer, homologous recombination deficiency, BRCA1/2, validation, next-generation sequencing

## Abstract

**Background:**

Along with *BRCA* mutation status, homologous recombination deficiency (HRD) testing is a prognostic and predictive biomarker for poly-ADP-ribose polymerase (PARP) inhibitor therapy indication in high-grade epithelial ovarian, fallopian tube, or peritoneal cancer. Approximately 50% of high-grade serous ovarian cancers exhibit HRD, even in the absence of germline or somatic *BRCA*1/2 loss-of-function mutations. In this scenario, access to a validated diagnostic HRD test can optimize treatment selection and increase the effectiveness of the intervention.

**Objective:**

To technically validate an *in-house* next-generation sequencing (NGS)-based HRD test, QIAseq Custom Panel (QIAGEN), by comparing it with the reference assay, MyChoice CDx® Plus HRD (Myriad Genetics), which is used in routine care.

**Methods:**

This is a prospective cohort study conducted at the Oncoclínicas Precision Medicine (OCPM) laboratory using samples from patients with advanced or relapsed platinum-sensitive ovarian cancer eligible for HRD testing in a diagnostic clinical setting at Oncoclínicas and Co. We assessed the performance of the in-house test (GS Focus HRD) using Cohen’s kappa statistic to measure agreement with the gold standard assay (MyChoice® HRD Plus CDx) in HRD status classification, along with other accuracy metrics.

**Results:**

In total, 41 samples were analyzed (20 HRD-positive, 19 HRD-negative, and 2 inconclusive results with the MyChoice® HRD Plus CDx assay). The GS Focus HRD test demonstrated high concordance for HRD status with the reference test (kappa: 0.8 and 95% CI: 0.60–0.98). Overall accuracy, sensitivity, and specificity were 90%. Six samples had *BRCA1*/*2* mutations identified by the MyChoice® HRD Plus CDx, all of which were detected by the GS Focus HRD test.

**Conclusion:**

In summary, the results demonstrate substantial agreement and high accuracy of the NGS-based GS Focus HRD test compared to MyChoice® HRD Plus CDx. Our in-house assay is eligible for diagnostic test approval and market access as per Brazilian regulations.

## 1 Introduction

With an estimated incidence of over 300,000 new cases per year, ovarian cancer (OC) is the eighth most common cancer in women worldwide and the third most frequent gynecological tumor (Bray et al., 2024). The most prevalent histological subtype is high-grade serous ovarian carcinoma (HGSOC), which is usually diagnosed at an advanced stage due to the lack of effective screening for early diagnosis (Torre et al., 2018; Dexter et al., 2024). Traditional treatment, consisting of cytoreductive surgery followed by platinum-based chemotherapy, achieves high rates of disease control; however, 60%–70% of patients will eventually experience recurrence (Ledermann et al., 2018). This scenario has undergone major changes since the advent of poly (adenosine diphosphate-ribose) polymerase inhibitors (PARPis), highly effective drugs for patients with *BRCA1/2* gene mutations and/or homologous recombination deficiency (HRD) ovarian cancer (Moore et al., 2018).

Homologous recombination deficiency represents a critical mechanism of genomic instability in cancer, characterized by impaired repair of DNA double-strand breaks through the homologous recombination repair (HRR) pathway. HRD significantly impacts the development and treatment of various cancers, such as ovarian, breast, pancreatic, and prostate cancers. For instance, a study analyzing 1,363 samples from various solid tumors found that an HRD-RNA model effectively predicted BRCA status in prostate and pancreatic cancers, with F1 scores of 0.88 and 0.69, respectively (Leibowitz et al., 2022). This highlights the importance of assessing HRD across multiple cancer types to inform treatment strategies, such as the use of PARP inhibitors, which are particularly effective in HR-deficient tumors (Stewart et al., 2022).

Although germline mutations in BRCA1 and BRCA2 are well-established contributors to HRD (Takaya et al., 2020; Vergote et al., 2022), this perspective is overly simplistic as a broader spectrum of genetic and epigenetic mechanisms underpins HRD. Mutations in additional HRR genes, such as ATM, PALB2, RAD51, and CHEK2, have emerged as significant drivers of HRD by disrupting various stages of the HRR pathway. These mutations highlight the complexity of DNA repair networks and their role in maintaining genomic stability. Furthermore, epigenetic alterations, particularly BRCA1 promoter methylation, further contribute to HRD by silencing the gene, resulting in functional deficiencies without the presence of genetic mutations. Loss of heterozygosity (LOH) is another hallmark of HRD, where the loss of the wild-type allele exacerbates defects associated with mutations or epigenetic silencing of HRR genes. Structural variations, including chromosomal rearrangements and large deletions, also play a pivotal role in altering HRR function, thereby contributing to the HRD phenotype. Importantly, HRD can arise not only from germline mutations but also from somatic mutations in HRR genes, underscoring the diverse origins of this phenotype. Somatic alterations broaden the clinical relevance of HRD as they can affect patients without a familial predisposition to cancer. The complexity of HRD mechanisms highlights the need to look beyond BRCA1/2 mutations to encompass a wider array of genetic, epigenetic, and structural variations. A comprehensive understanding of these mechanisms is crucial for accurate HRD assessment and for optimizing therapeutic strategies, such as the use of PARP inhibitors and other precision oncology approaches in HR-deficient tumors (Mekonnen et al., 2022).

Nearly half of the women with HGSOC have HRD, while only approximately 21% of the patients carry germline or somatic *BRCA1/2* loss-of-function mutations. In this scenario, diagnostic tests to assess tumor HRD status are validated predictive biomarkers for PARPi therapy in ovarian cancer (Vergote et al., 2022). Therefore, access to a validated HRD test can optimize treatment selection and increase the effectiveness of the intervention. In multiple studies, MyChoice® CDx genomic instability score (GIS) (Myriad Genetics) has been used to determine HRD status (Myriad Genetic Laboratories and Inc., 2019). This test evaluates ‘genomic scars’ that serve as a surrogate measure of HRD as they represent a footprint of genomic changes induced by DNA repair deficiency. Specifically, the gold-standard HRD genomic scar assay evaluates the percentage of genomic regions with LOH, telomeric allelic imbalance (TAI), and large-scale transitions (LSTs) in a combined GIS. This test also provides information on *BRCA1/2* mutation status (González-Martín et al., 2019; Ray-Coquard et al., 2019). Another test to evaluate LOH score and *BRCA1/2* mutation status is the FoundationOne® CDx (Foundation Medicine Inc., Cambridge, MA, United States) (Frampton et al., 2013). Although it has been used in phase 3 studies with PARP inhibitors, this test does not assess the GIS signature like the reference MyChoice® CDx (Monk et al., 2022).

Many laboratories are developing in-house HRD tests using comparable methods to identify GIS signatures, and the results to date point to high concordance rates (Fountzilas et al., 2023; Fumagalli et al., 2022; Guarischi-Sousa et al., 2023). Knowledge in this field is evolving rapidly, and there is a critical need to technically and clinically validate alternative HRD tests so that more patients can have access to a robust biomarker to guide treatment decisions in ovarian cancer.

The present study aimed to assess the agreement rate of next-generation sequencing (NGS)-based HRD assay, QIAseq Custom Panel (QIAGEN), hereafter referred to as GS Focus HRD, with the gold-standard HRD test, Myriad MyChoice® CDx (Myriad Genetics), which is used in clinical practice.

## 2 Materials and methods

### 2.1 Patient cohort

This is a prospective cohort study conducted at the Oncoclínicas Precision Medicine (OCPM) Molecular Pathology and Genomics Laboratory to technically validate the GS Focus HRD test. Approval was obtained from the Institutional Review Board (IRB) and the National Committee of Ethics in Research (CONEP), a Brazilian entity that evaluates the ethical aspects of research involving human beings, under Protocol CAAE: 67821223.3.0000.0227. All subjects consented to participate in the study.

Tissue samples from patients with advanced or relapsed platinum-sensitive ovarian cancer treated at Oncoclínicas and Co. outpatient clinics were collected as part of routine care for HRD testing. The same formalin-fixed paraffin-embedded (FFPE) tumor block was sent to MyChoice® HRD Plus CDx and analyzed in-house with GS Focus HRD. Although the test evaluated mutations in HRR genes, the orthogonal validation consisted of GIS score assessment (LOH/TAI/LST) along with somatic *BRCA1/2* mutation status to define a tumor as HRD (if GIS score ≥ 42 or *BRCA1/2* mutated) or homologous recombination proficient (HRP, if GIS <42 and *BRCA1/2* wild-type). Samples were analyzed by the central Myriad Genetics Laboratory (United States) through an established partnership with GenCell Laboratory (Brazil).

### 2.2 DNA extraction and NGS

To perform GS Focus HRD, appropriate FFPE tissue was defined as containing >20% tumor cells and <10% necrosis, as determined by the local laboratory’s pathologist. DNA was extracted using the ReliaPrep FFPE System (Promega), according to the manufacturer’s protocol. NGS library preparations for the QIAseq Custom Panel (QIAGEN) were performed according to the manufacturer’s recommendations, considering a minimum DNA input of 100 ng.

### 2.3 Bioinformatics analyses

The GS Focus HRD panel analyzed 13,809 single nucleotide polymorphisms (SNPs) to detect LOH, TAI, and LST, with the GIS representing the sum of these events. As indicated by the manufacturer (QIAGEN), the test is reported as HRD or “positive” status when the GIS is ≥65; otherwise, the test is reported as HRP or “negative” status. In addition, the test was designed to provide information on single nucleotide variants (SNVs) and indels in 15 homologous recombination repair (HRR) genes: *BRCA1*, *BRCA2*, *ATM*, *CHEK2*, *PALB2*, *BRIP1*, *FANCA*, *RAD51B*, *RAD51C*, *RAD51D*, *CDK12*, *RAD54L*, *FANCL*, *CHEK1*, and *BARD1*. Sample preparation, sequencing, and bioinformatic analysis were conducted at the OCPM laboratory. Sequencing data (paired-end reads 2 × 150) were analyzed using CLC Genomics Workbench (QIAGEN) using the pipeline developed by the manufacturer for both genomic scar detection (GIS) and variant calling.

### 2.4 Comparative analyses

For significant agreement between two classifiers using the kappa statistic, 40 patients were required. This sample size achieves 80% power at a significance level of 0.05 to detect a true kappa value of 0.85 (near complete concordance). We estimated the prevalence of HRD to be at least 40% using the gold standard assay.

The results of both tests were aggregated into the study database for comparative analysis. Statistical analysis was performed by calculating the concordance (Cohen’s kappa). We also assessed the overall accuracy, sensitivity, and specificity of the GS Focus HRD test compared to MyChoice® HRD Plus CDx. In addition, we assessed the correlation of GIS scores between the two assays using R-squared statistics.

## 3 Results

A total of 41 patients with HGSOC eligible for HRD testing in routine care were recruited into the study, as listed in Table 1.

**TABLE 1:** Agreement analysis of the HRD score between GS Focus HRD and the MyChoice® HRD Plus CDx test.

Patient ID	GS focus HRD score (cutoff: 65)	GS focus HRD status	MyChoice® HRD score (cutoff: 42)	Myriad HRD status	Concordance
2169053	74	Positive	65	Positive	True
2101839	0	Negative	0	Negative	True
2389540	34	Negative	31	Negative	True
2381018	91	Positive	52	Positive	True
2293728	100	Positive	73	Positive	True
2301111	55	Negative	27	Negative	True
2671076	63	Negative	64	Positive	False
2210673	59	Negative	29	Negative	True
2380324	73	Positive	73	Positive	True
2474920	59	Negative	47	Positive	False
2671537	45	Negative	23	Negative	True
2678279	90	Positive	46	Positive	True
2370979	78	Positive	73	Positive	True
2931692	16	Negative	6	Negative	True
2670650	60	Negative	12	Negative	True
2483006	52	Negative	16	Negative	True
2276787	64	Negative	23	Negative	True
2483066	115	Positive	73	Positive	True
2317041	64	Negative	39	Negative	True
2614298	40	Negative	33	Negative	True
2315525	45	Negative	36	Negative	True
2344032	32	Negative	11	Negative	True
2491947	75	Positive	31	Negative	False
2251070	58	Negative	Inconclusive	Inconclusive	-
2389417	86	Positive	57	Positive	True
2101053	60	Negative	20	Negative	True
2312229	69	Positive	50	Positive	True
2221210	90	Positive	63	Positive	True
2344268	84	Positive^a^	81	Positive^a^	True
2055315	43	Positive^a^	49	Positive^a^	True
2067067	56	Positive^a^	49	Positive^a^	True
2362389	101	Positive	71	Positive	True
2507049	23	Negative	6	Negative	True
2419258	77	Positive	48	Positive	True
2447558	89	Positive	70	Positive	True
2304821	68	Positive^a^	65	Positive^a^	True
2025663	17	Negative	Inconclusive	Inconclusive	-
2418680	64	Negative	22	Negative	True
2199981	96	Positive^a^	Inconclusive	Positive^a^	True
2351654	76	Positive	33	Negative	False
2230224	48	Negative	12	Negative	True

^a^ Positive *BRCA1*/*2* mutation status.

As shown in Tables 1, 2, 20 samples were HRD-positive, 19 were HRD-negative (HRP), and 2 (R01Q11 and R01Q01) had inconclusive results with the MyChoice® HRD Plus CDx assay, and for this reason, they were removed from the concordance analysis. As per the GS Focus HRD test, 20 samples were HRD-positive, 21 HRD-negative (HRP), and none had inconclusive results.

**TABLE 2:** Concordance analysis of HRD status between the 41 samples analyzed by the GS Focus HRD test and MyChoice® HRD Plus CDx. Inconclusive cases were reported when the result could not be provided.

GS focus HRD status	MyChoice® HRD plus CDx assay	
	HRD status positive	Negative
Positive	18	2
Negative	2	17
Inconclusive		
MyChoice® HRD Plus CDx assay	2	
GS Focus HRD assay	0	

**FIGURE 1:**
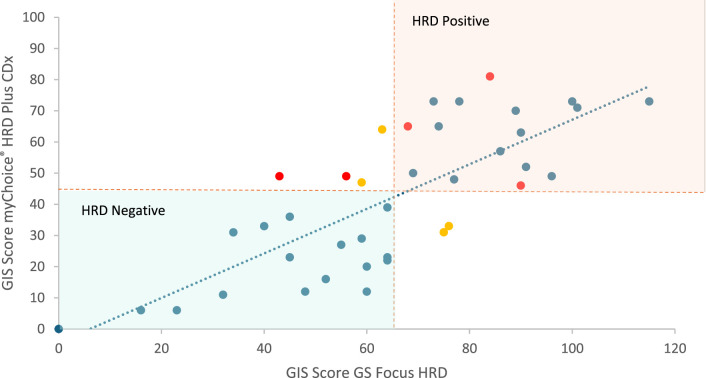
Correlation between GIS scores obtained by both methodologies. The dotted lines indicate the cutoff values for the MyChoice® HRD Plus CDx (42) and GS Focus HRD (65) tests, respectively. Red dots indicate samples with oncogenic alterations in *BRCA1/2*. Yellow dots indicate discordant samples between the two tests performed. Blue dots indicate samples with no pathogenic mutations identified in *BRCA1/2* genes. HRD-positive status samples corresponded to those enclosed in the red area, while HRD-negative status samples corresponded to those enclosed in the blue area.

A total of 5 out of 39 samples had pathogenic *BRCA1*/*2* mutations (13%) identified by both assays (indicated as red dots in Figure 1), while 37 samples had no pathogenic mutation reported in BRCA1/2.

Overall, the GS Focus HRD test demonstrated high agreement with the reference test for HRD status (kappa: 0.8 and 95% CI: 0.60–0.98), excluding two samples with inconclusive MyChoice® HRD Plus CDx assay results. One sample was reported by Myriad as inconclusive due to the insufficient quality or quantity of DNA. The second case was reported as inconclusive due to the inability to analyze GIS status. However, HRR including BRCA1/2 mutation status was provided. We can consider the inconclusive rate in this cohort to be 5%, similar to the study reported by Capoluongo et al. (2022) (6% in a cohort of 100 patients) and close to another study that reported a 9% inconclusive rate for the Myriad test in a cohort of 469 patients (Christinat et al., 2023).

Considering GIS status concordance, samples (10%) exhibited genuine disagreements: R04Q04 and R03Q22 were considered HRD-negative by GS Focus HRD (scores of 63 vs. 64 and 59 vs. 47, respectively) but HRD-positive by MyChoice® HRD Plus CDx. On the other hand, R03Q19 and R03Q11 were considered HRD-positive by GS Focus HRD (scores of 75 vs. 31 and 76 vs. 36, respectively) but HRD-negative by MyChoice® HRD Plus CDx. As shown in Figure 1, the GIS scores of the discordant samples were close to the cutoff for HRD/HRP classification defined by the manufacturer. In addition, two samples (R01Q02 and R01Q03) harbored pathogenic variants in *BRCA1*/*2* with GIS scores of 49 as per MyChoice® HRD Plus CDx (classified as HRD). Although the GIS score of these two cases was <65 as per the GS Focus HRD assay, they were classified as HRD-positive based on the positive *BRCA1*/*2* mutation.

Next, we evaluated the correlation between the GIS scores obtained by both methodologies (Figure 1). The two methodologies analyzed in this study have different thresholds or cutoff values (65 for QIAGEN and 42 for Myriad) to define HRD status, so we do not expect a complete correlation between HRD scores. However, we observed a decent correlation (R squared = 62), which agrees with the good concordance in terms of HRD status.

Overall, the test has a sensitivity, specificity, and accuracy of 90% with respect to the gold standard (Table 3).

**TABLE 3:** Performance analysis for the GS Focus HRD test.

Statistic	Value (%)	95% CI (%)
Sensitivity	90.91	70.84–98.88
Specificity	90.00	68.30–98.77
Positive predictive value	90.91	72.73–97.40
Negative predictive value	90.00	70.43–97.14
Accuracy	90.48	77.38–97.34

## 4 Discussion

The prognosis of patients with advanced ovarian cancer has dramatically improved due to recent advancements in precision oncology, especially the use of targeted drugs, which have altered the therapeutic landscape. The incorporation of HRD assessment, beyond *BRCA1/2* mutation status, as a significant biomarker for therapeutic decisions, presents a substantial challenge in clinical practice.

HRD is present in approximately 50% of HGSOC and is predictive of the efficacy of PARP inhibitors (PARPis). It can be detected through two molecular strategies. The first approach identifies the underlying genetic causes of HRD, while the second evaluates the tumor phenotype by assessing genomic instability. Although expanding NGS panels to incorporate HRR genes beyond *BRCA1/2* could only improve the detection rate of tumors with HRD by 5%–6% (Guarischi-Sousa et al., 2023), the FDA-approved MyChoice® HRD Plus CDx assay remains the market reference due to its clinical validation. This assay assesses both *BRCA1/2* status and HRD-induced genomic scarring. However, despite its significance, MyChoice® HRD Plus CDx is centrally performed, costly, and not covered by health insurance companies, rendering it inaccessible to many patients (González-Martín et al., 2019).

In response to the clinical demand for more efficient, accurate, and rapid alternatives, numerous novel assays have been developed recently to evaluate HRD status. However, the implementation of these tests in clinical practice may be hindered by variations in methodology and confusion regarding the measurement and reporting of HRD status. Importantly, HRD tests are complex genomic signatures that can yield non-informative results when DNA is extracted from paraffin blocks of poor quality, often due to pre-analytical parameters associated with inadequate preparation or preservation. Failure rates of up to 25% have been reported in real-world HRD testing (Myriad Genetic Laboratories and Inc., 2019). Therefore, the performance of novel HRD tests must be assessed and contextualized based on the local standards of tissue quality. Many medical centers have attempted to utilize in-house HRD testing to simplify technical processes, workflows, and data interpretation.

In this report, we describe our experience with the QIAseq Custom Panel (QIAGEN) (GS Focus HRD) in the diagnostic workflow, focusing on the feasibility and reliability of in-house HRD testing compared to the gold-standard HRD test, MyChoice® HRD Plus CDx (Myriad Genetics), which is utilized in routine care. Overall, GS Focus HRD demonstrated a sensitivity and specificity of 90% compared to the gold standard. The agreement rate had a Cohen’s kappa of 0.8 (95% CI: 0.60–0.98), indicating substantial to near-perfect agreement.

Our findings are consistent with previous studies that have compared various HRD assays with the MyChoice® HRD Plus CDx assay, despite the discordance found in GIS status. For instance, the AmoyDx® HRD Focus Panel exhibited a high concordance rate of 87.8%, with 65 out of 74 tumors evaluated showing concordant HRD results (Konstantinopoulos et al., 2015). Another study reported 100% concordance in HRD status between the AmoyDx® HRD Focus Panel and MyChoice® HRD Plus CDx assays in a cohort of only 13 patients with epithelial ovarian cancer (Magliacane et al., 2022). Furthermore, other preliminary studies comparing HRD status across the AmoyDx® HRD Focus Panel, OncoScan™, and MyChoice® HRD Plus CDx assays have shown substantial concordance among the three tests, with Cohen’s kappa values exceeding 0.75 for all comparisons (Weichert et al., 2021). The ENGOT European HRD Initiative developed the “Leuven” HRD test using ovarian cancer tumor tissue, aiming to validate a laboratory-developed HRD assay comparable to the MyChoice® HRD Plus CDx assay. This test was used to assess the HRD status of 468 ovarian tumor samples from the PAOLA-1/ENGOT-ov25 trial, revealing a 91% overall agreement with the MyChoice® HRD Plus CDx assay, with positive and negative percent agreements of 94% and 86%, respectively (Loverix et al., 2022). A recent study from Brazil evaluated the performance of two commercial kits, SOPHiA DDM™ HRD Solution and the AmoyDx® HRD Focus Panel, in comparison to the reference MyChoice® HRD Plus CDx for in-house HRD testing. The study found a significant association among the three assays, despite variations in the methodologies employed to assess genomic instability in tumor samples. The strongest correlation was observed between MyChoice® HRD Plus CDx and SOPHiA DDM™, with SOPHiA DDM™ achieving a positive predictive value (PPV) of 90.0% and a negative predictive value (NPV) of 96.3% (Guarischi-Sousa et al., 2023).

Our study presents limitations that may impact the interpretation of its findings. First, although the sample size was sufficient to address the primary study hypothesis, a larger cohort could help define a “custom” cutoff for HRD positivity with our assay in the laboratory, thereby improving the overall performance further. A significant limitation is the quality of the tumor samples from which DNA is extracted, as suboptimal samples can result in inconclusive outcomes, thereby influencing clinical decisions. Moreover, using the same NGS data or DNA for both analyses could help eliminate variability and provide more robust comparisons to evaluate the accuracy of NGS bioinformatics. Although the novel assay evaluated in this study offers advantages in terms of efficiency and potentially reduced turnaround time, its associated costs relative to traditional methods may still pose substantial barriers to implementation, particularly in low-resource settings. We estimate that local testing could be 30%–40% less expensive than the gold standard, offering significant cost-effectiveness and potentially increasing accessibility to broader patient populations, especially in resource-limited settings, while maintaining comparable diagnostic accuracy. These factors should be considered when assessing the overall practicality of HRD testing in clinical settings.

## 5 Conclusion

In summary, the results demonstrate substantial agreement and high accuracy of the NGS-based GS Focus HRD test compared to MyChoice® HRD Plus CDx. Our in-house assay is eligible for diagnostic test approval and market access as per Brazilian regulations.

## Data Availability

The raw data supporting the conclusion of this article will be made available by the authors upon request.
